# Holistic Investigation of Graphene Quantum Dot Endocytosis

**DOI:** 10.1002/smll.202406095

**Published:** 2025-02-02

**Authors:** Ugur C. Topkiran, Alina R. Valimukhametova, Diya Vashani, Himish Paul, Abby Dorsky, Olivia Sottile, Dustin A. Johnson, William Burnett, Jeffery L. Coffer, Giridhar R. Akkaraju, Anton V. Naumov

**Affiliations:** ^1^ Department of Physics and Astronomy Texas Christian University TCU Box 298840 Fort Worth TX 76129 USA; ^2^ Department of Chemistry and Biochemistry Texas Christian University TCU Box 298840 Fort Worth TX 76129 USA; ^3^ Department of Biology Texas Christian University TCU Box 298840 Fort Worth TX 76129 USA

**Keywords:** graphene quantum dots, nanocarbons, nanoparticle endocytosis, nanoparticle trafficking

## Abstract

Graphene quantum dots (GQDs) have gained popularity in nano‐biotechnology due to their multifunctional delivery and imaging capabilities. The outcome of their therapeutic delivery applications relies on understanding cell internalization routes. Current literature presents often conflicting results based on surveying only a few endocytosis inhibitors. Herein, a holistic approach to cell uptake studies by utilizing six different inhibitors while considering their on‐ and off‐target effects on internalization of the GQDs of different charges is provided. Endocytosis paths are explored by tracking intracellular GQD fluorescence in HeLa or HEK‐293 cells. Contrary to the previous assumptions of a singular entry route, findings suggest that GQDs enter the cells through several endocytosis paths with some more prevalent than others. Selectivity between the pathways is based on GQD charge and functional groups. Positively charged nitrogen‐doped GQDs (NGQDs) predominantly utilize a fast endophilin‐mediated endocytosis (FEME) in HeLa cells with a secondary preference for clathrin‐mediated endocytosis (CME). In HEK‐293 cells NGQDs internalize via clathrin‐independent, glycosylphosphatidylinositol‐anchored protein‐enriched compartments (CLIC/GEEC) and FEME. Conversely, GQDs with a substantial negative surface charge uptake through CME in HeLa cells. The optimization of these mechanisms can enhance GQD applications in biomedicine, ideally streamlining their translation into the clinic.

## Graphical abstract

Six chemical endocytosis inhibitors are used to selectively block and analyze preferential uptake pathways of Graphene Quantum Dots (GQDs) in cancerous (HeLa) versus non‐cancerous (HEK‐293) cells via fluorescence microscopy. Contrary to previously perceived singular mechanism for GQD endocytosis, this work suggests that GQDs prefer a combination of multiple pathways depending on their surface charge and functional groups.

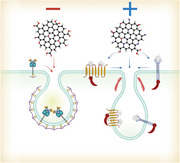

## 1 Introduction

The use of nanoparticles, as drug delivery vehicles to enhance the treatment of genetic disorders and cancer therapy, has increased substantially within the last decade.^[^ ^1^ ^]^ The flexibility of synthetic design as well as the nanoscale form factor allows nanoparticles to effectively serve as imaging,^[^ ^2^ ^]^ sensing,^[^ ^3^ ^]^ and delivery agents that can be augmented by receptor targeting capabilities.^[^ ^4^ ^]^ A variety of nanoparticles have already been tested *in vitro* and *in vivo* including liposomes, polymeric nanoparticles, 2D nanomaterials, inorganic gold, iron, silver, and silica nanoparticles as well as inorganic quantum dots, some of which have already advanced to clinical trials.^[^ ^1^, ^5^, ^6^ ^]^

Another novel class of materials, nanocarbons, including fullerenes, carbon nanotubes (CNTs), graphene oxide (GO), and GQDs carries promising potential in drug delivery applications due to their versatility of functional forms as well as remarkable physical, optical, and electronic properties and the possibility of covalent and non‐covalent functionalization with the variety of therapeutic agents.^[^ ^7^ ^]^ Augmenting nanocarbon materials non‐covalently with nucleic acids allows applications in gene delivery, whereas conjugating them with targeting agents, such as folic acid, improves their binding to target receptors for enhanced accumulation at the disease site.^[^ ^8^ ^]^ Complexation of such nanocarbon delivery vehicles with partly hydrophobic therapeutics can ensure their successful delivery.^[^ ^9^ ^]^ In addition to targeting and drug delivery, nanocarbons can also facilitate therapeutic tracking and analyte detection via their intrinsic fluorescence. For instance, semiconducting single‐walled carbon nanotubes (SWCNTs) exhibit NIR emission,^[^ ^10^ ^]^ beneficial for bioimaging due to minimized scattering^[^ ^11^ ^]^ and autofluorescence in that spectral region ensuring high tissue penetration.^[^ ^12^ ^]^ Their NIR fluorescence allows SWCNTs to be utilized for *in vitro* and *in vivo* therapeutic applications. These include the tracking of cancer therapeutics such as doxorubicin and paclitaxel, and gene therapies including short interfering RNA and plasmid DNA.^[^ ^13^ ^]^ However, further advancement of such an advantageous delivery platform is hampered by an apparent dependence of SWCNT biocompatibility on surfactant coating, which can be displaced by biological molecules. Another water‐soluble nanocarbon candidate for drug delivery and intrinsic fluorescence‐facilitated imaging is GO.^[^ ^14^ ^]^ However, the level of delivered therapeutic doses for GO is also restricted by its toxicity at higher concentrations and substantial long‐term tissue accumulation.^[^ ^15^ ^]^

These issues can be addressed with a novel class of carbon nanomaterials, GQDs. The number of studies and publications addressing the biomedical uses of GQDs has experienced an exponential increase over the past decade due to their remarkable properties that can be tuned to satisfy different biomedical applications.^[^ ^16^ ^]^ Being 0D sub‐20 nm nanoparticles, GQDs manifest the highest biocompatibility within the nanocarbon family, which has already been demonstrated both *in vitro* and *in vivo*.^[^ ^17^, ^18^ ^]^ They can be synthesized either using a “top‐down” method of chemically exfoliating larger graphene derivatives, or a “bottom‐up” approach, involving hydrothermal carbonization of molecular precursors. GQD structure allows for several versatile ways of attaching therapeutics and targeting agents either via π‐π stacking on top of their graphitic surface, electrostatic interactions with GQD functional groups, or covalent functionalization to those. These mechanisms have been successfully utilized in delivering chemotherapeutics,^[^ ^19^ ^]^ genes such as plasmid DNA, chimeric peptides, short interfering RNA,^[^ ^20^, ^21^ ^]^ and CRISPR‐Cas 9 RNP gene therapy.^[^ ^22^ ^]^ GQDs exhibit highly photostable intrinsic fluorescence in the visible originating from size‐dependent quantum confinement.^[^ ^23^ ^]^ Several GQD structures have recently been developed to exhibit fluorescence in the NIR originating from defect states at multiple functional groups.^[^ ^24^, ^25^ ^]^ Their fluorescence properties have paved the way for GQD‐based optical sensors detecting proteins,^[^ ^26^ ^]^ RNA,^[^ ^27^ ^]^ micro RNA,^[^ ^28^ ^]^ single‐stranded DNA,^[^ ^29^ ^]^ and small molecules.^[^ ^16^ ^]^ GQDs can be synthesized scalably and cost‐effectively in large quantities suitable for extensive drug screening studies. They exhibit rapid cellular internalization and excretion from the cells or organs^[^ ^17^ ^]^ unlike many other nanomaterials residing in biological tissues for prolonged periods.

Owing to the aforementioned advantages, GQDs are becoming one of the most actively developing drug delivery platforms for *in vitro/in vivo* applications. However, despite widespread use, their internalization mechanism has not been sufficiently studied. Due to their size, in the range of 3 nm, it is expected that their cellular uptake occurs via endocytosis.^[^ ^30^, ^31^ ^]^ Endocytosis plays an extensive role in such cellular functions as the uptake of nutrients, cell‐surface homeostasis, synaptic transmission, immune responses and can be carried out via several distinctly different pathways.^[^ ^32^ ^]^ Although receptor coated pits only occupy ≈2% of the cell membrane, they internalize an area equal to that of the whole membrane every two hours.^[^ ^33^, ^34^ ^]^ This facilitates efficient internalization of larger cargo, such as nanomaterials, making endocytosis an effective primary pathway for the uptake of larger entities despite the lower overall percentage occupancy of receptors on the cellular membrane. Lack of successful targeting of these entry pathways may facilitate the activation of cellular defense mechanisms such as clearance of the non‐internalized nanomaterials by the mononuclear phagocytic system (MPS).^[^ ^35^ ^]^ Thus, a thorough understanding of nanoparticle entry mechanisms can help prevent triggering undesirable processes, hampering the transition of nanomedicines to the clinic. Endocytosis is initiated when a nanoparticle first interacts with the cell membrane and the outcome of this interaction is dependent on the functional groups present on the surface, the charge, the size and the shape of the nanoparticle. To clarify, a sheet‐like nanomaterial, such as GO, would go through different endocytosis paths depending on the lateral flake size.^[^ ^36^ ^]^ As a result, CME is expected for the uptake of smaller flakes, while larger‐sized GO structures are claimed to be uptaken by phagocytosis.^[^ ^36^ ^]^ Other mechanisms can also participate in facilitating nanomaterials’ entry. A dynamin‐dependent path, CME, is known to harbor nanoparticles by forming vesicles up to ≈100 nm in size setting the upper size limit for their internalization.^[^ ^37^ ^]^ Another dynamin‐dependent but clathrin‐independent path, known as FEME, facilitates extremely rapid entry (<10s). During this process tube‐like vesicles of 60 nm in diameter and several hundred nanometers in length form on the cell membrane enabling nanomaterial trafficking.^[^ ^38^ ^]^ Conversely to CME and FEME, another feasible pathway for nanomaterial uptake is clathrin‐independent/dynamin‐independent endocytosis that is mediated by clathrin‐independent vesicles arising from the plasma membrane and maturing into tubular endocytic compartments named CLIC/GEEC.^[^ ^39^ ^]^ It is observed to be a distinct high‐capacity pathway in mammalian cultured cells, only sharing similarities with FEME through their localization to the edge of migrating cells and involvement of ring‐shaped tubular pleomorphic vesicles. Extra‐cellular mechanism in clathrin independent uptake is operated through galectin‐3 lectin proteins that trigger the formation of the CLIC/GEEC vesicles.^[^ ^40^ ^]^ While FEME is stimulated through specific ligand‐receptor interactions, CLIC/GEEC shows continuous uptake without the need for a receptor trigger.^[^ ^41^ ^]^ Caveolin‐mediated endocytosis (Cav) is limited by its distinct morphology, which presents a bulb‐shaped pit of 60 nm in diameter attached to the plasma membrane by a slightly smaller neck.^[^ ^42^ ^]^ As a consequence of the bulb's size, Cav is known to accommodate smaller particles of sizes ≈50 nm or less. Macropinocytosis (Macro) and phagocytosis (Phago) are two of the most studied dynamin‐independent but actin‐dependent pathways that uptake particles with a size larger than 200 nm. Even though the formation of the phagocytosis vesicles is triggered by scavenger receptors, GQD sizes are below the Phago uptake limit, which makes scavenger receptor‐mediated GQD endocytosis unlikely.^[^ ^43^ ^]^ Furthermore, certain endocytosis pathways are lipid raft‐dependent: lipid raft's complex structure can allow the uptake of the same cargo through multiple endocytosis pathways.^[^ ^44^ ^]^ Dynamin, that is present in both CME and FEME is shown to be an indicator of lipid raft‐dependency of an endocytosis pathway.^[^ ^45^ ^]^ However, Cav, a dynamin‐ and clathrin‐independent pathway is also observed to be lipid raft‐dependent.^[^ ^46^ ^]^ In the case of SWCNTs, internalization proceeds through energy‐dependent endocytosis, such as CME, Cav, and Macro depending on their length.^[^ ^47^ ^]^ Endocytosis is observed to be the main internalization route for SWCNTs since they lack the sufficient insertion energy to penetrate the plasma membrane of a cell via nanospearing.^[^ ^48^ ^]^ SWCNT association with lipids alters the membrane tension, therefore stimulating endocytosis on the membrane. While such membrane diffusion is demonstrated to be the main form of cellular uptake for single‐layer GQDs of sizes ranging from 2 nm.,^[^ ^49^ ^]^ there are no sufficient studies displaying membrane diffusion uptake of multi‐layer GQDs. Previous endocytosis studies on carbon‐based nanomaterials were performed with larger graphene oxide or carbon oxide particles of sizes ranging from 100 to 300 nm. Those show internalization through a combination of CME and Cav, suggesting multiple pathways can be utilized for large nanocarbons.^[^ ^50^, ^51^, ^52^, ^53^ ^]^ GO flakes of sizes ranging from 200 to 300 nm are also reported to utilize CME whereas smaller flakes of sizes ∼100 nm internalize through Cav.^[^ ^54^ ^]^ However, being smaller and having a different combination of surface functional groups, GQDs present a novel platform to be studied. Given a variety of different internalization mechanisms associated with other carbon nanomaterials, it is hard to predict that of the GQDs, especially given the scarcity of such studies for this novel material. An understanding of the GQDs’ endocytosis process will provide greater insights into nanoparticle/cell membrane interactions that can help increase internalization efficiency and avoid cell defense mechanisms. As different cell types possess different endocytosis paths, understanding the GQD uptake mechanism can also aid in targeting specific cells for enhanced drug delivery.

Previously, Wang et. al proposed that hollow carbon dots synthesized from bovine serum albumin delivering doxorubicin enter the A549 cells by forming vesicles further fused with lysosomes attributing the uptake to endocytosis.^[^ ^55^ ^]^ Studies done on human neural stem cells unveiled that GQD uptake occurs in a time and concentration‐dependent manner.^[^ ^56^ ^]^ Later works claimed that GQDs mainly prefer endocytosis by Cav in MCF‐7 and MGC‐803^[^ ^57^ ^]^ cell lines and CME in HUVEC and HeLa^[^ ^58^ ^]^ cell lines as well as in *Trypanosoma brucei*.^[^ ^59^ ^]^ These conclusions were drawn based on the substantial decrease of intracellular GQD fluorescence upon cell treatment with corresponding pathway inhibitors. Importance of a holistic approach that considers the possibility nanomaterial internalization through multiple pathways has previously been suggested and is displayed for other nanomaterials.^[^ ^60^, ^61^ ^]^ Moreover, some GQD endocytosis inquiries made by Xu et al. consider a combination CME and Cav pathways.^[^ ^62^ ^]^ However, these approaches do not provide a holistic understanding in their consideration of the variety of other potential uptake pathways for the GQDs. Different – and often conflicting – results presented by current works arise from surveying only a few inhibitors and disregarding potential off‐target pathway inhibition (**Table** **1**). This can lead to attributing the entry of sub‐10 nm GQDs to Macro or Phago,^[^ ^63^, ^64^ ^]^ while these mechanisms favor larger 200 nm to 0.5 µm particle sizes and are very unlikely to explain the uptake of GQDs. The effects of different GQD surface charges, functional groups, and certain receptor‐ligand interactions within the endocytosis pathways also require consideration.

The present work introduces a holistic approach to the assessment of potential GQD entry pathways to address these factors. Entry pathways of different GQD structures with varying surface charges will be evaluated based on GQD samples hydrothermally bottom‐up synthesized from glucosamine precursor^[^ ^24^ ^]^ and GQDs top‐down‐derived from reduced graphene oxide.^[^ ^65^ ^]^ As a way to evaluate and, potentially, utilize endocytosis pathway differences between non‐cancer and cancer cells, GQD internalization is studied in this work in model cancer (HeLa) and non‐cancer (HEK‐293) cell lines with six of the most commonly used endocytosis path inhibitors; cytochalasin D, genistein, filipin, amiloride, chlorpromazine and sodium azide. These studies considering on‐ and off‐target inhibitor effects on top of their mechanisms of action provide a comprehensive fundamental understanding of the uptake paths of the GQDs, a material that is now increasingly used in nanomedicine applications including targeted drug delivery focused on specific cell entry pathways.

## 2 Results and Discussion

### 2.1 Properties of N‐ and RGQDs: Does Size and Charge Matter?

**Figure 1:**
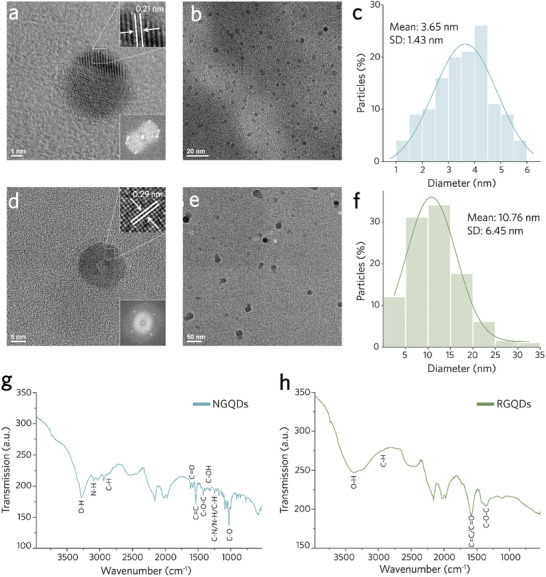
a) HRTEM images of NGQDs, d) RGQDs with insets displaying the interlayer spacings and FFT. b) TEM images of NGQDs, e) RGQDs revealing the size distributions. c) Nanoparticle size distribution histogram of NGQDs (blue), f) RGQDs (green); the mean and standard deviation of the distributions are indicated within the plots. g) FTIR spectra of NGQDs (blue), h) RGQDs (green); the peaks are marked with the corresponding functional groups present within the structure.

The internalization of two types of GQDs is studied in the present work: NGQDs bottom‐up synthesized from a glucosamine precursor and RGQDs synthesized top‐down from reduced graphene oxide. Both GQD types are thoroughly characterized to evaluate their physical properties affecting internalization. Transmission Electron Microscopy (TEM) images reveal that NGQDs and RGQDs have size distributions with average diameters of 3.65 ± 1.43 nm and 10.76 ± 6.45 nm respectively (**Figure** **1**c,f). Although there are size differences between NGQDs and RGQDs, both nanomaterials’ sizes are below the smallest endocytosis vesicle. Therefore, GQD sizes are not expected to substantially affect their internalization through the endocytosis paths studied, allowing their comparative studies. Both GQDs display graphitic lattices with planar spacings of 0.21 nm for NGQDs and 0.29 nm for RGQDs demonstrated by HRTEM, while their crystallinity is further confirmed by Fast Fourier Transform (FFT) images (Figure 1a,d insets). Furthermore, NGQD and RGQD spherical structures and 1 and 5 nm heights are verified through their AFM height profile analysis (Figure S1, Supporting Information). Fourier Transform Infrared Spectra (FTIR) of freeze‐dried samples indicate the presence of hydroxyl and carboxyl groups on their surface while NGQDs also exhibit amine group transitions indicative of successful nitrogen doping (Figure 1g,h). Both GQDs possess common bands of O‐H,^[^ ^66^ ^]^ C‐H,^[^ ^67^ ^]^ C = O (of COOH group) and, C‐O‐C^[^ ^68^ ^]^ at ≈ 3300 cm^−1^, ≈2932 cm^−1^, 1590 cm^−1^, and 1400 cm^−1^ respectively. The C = C stretch resides at ≈1530 cm^−1^ for NGQDs and at ≈1580 cm^−1^ for RGQDs, overlapping with its C = O band (Figure 1g,h).^[^ ^69^ ^]^ NGQD FTIR spectra also reveal N‐H, C‐OH, C‐N/N‐H/C‐H, and C‐O bands at ≈3090 cm^−1^, ≈1330 cm^−1^, ≈1246 cm^−1^, and ≈1028 cm^−1^ (Figure 1g) suggesting the presence of the corresponding functional groups.^[^ ^66^ ^]^ Moreover, X‐ray Photoelectron Spectroscopy (XPS) studies on NGQDs and RGQDs further confirm the presence of hydroxyl, carboxyl, and amine surface functional groups for both nanomaterials (Figures S2 and S3, Supporting Information). The potential of nanoparticle surface charges to initiate specific endocytosis pathways is considered in this work and surface charges of both GQDs are examined via ζ‐potential measurements at pH 7 (Figure S4, Supporting Information). NGQDs display a positive ζ‐potential of +1.8 ± 0.7 mV, likely due to the presence of amine groups, whereas RGQDs show a significantly more negative ζ‐potential of ‐49.4 ± 0.4 mV, attributed to the abundance of hydroxyl and carboxyl groups on their surface.

**Figure 2:**
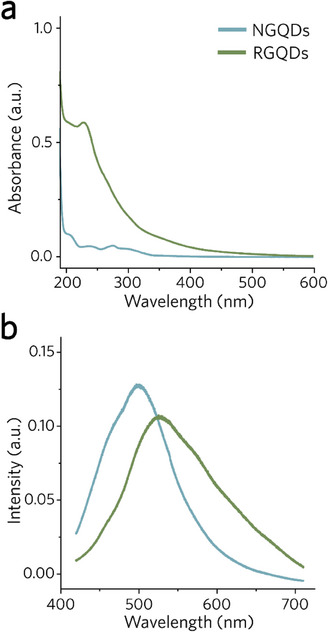
a) Absorbance spectra of NGQDs (blue) and RGQDs (green). b) Visible fluorescence spectra of NGQDs and RGQDs under 400 nm laser excitation.

Endocytosis studies are conducted with the aid of visible fluorescence microscopy; thus, both absorption and fluorescence spectra of both GQD types are analyzed prior to internalization experiments. Both NGQDs and RGQDs exhibit dominant absorption features ≈230 nm, attributed to π‐π* electronic transitions of C = C (**Figure** **2**a). An absorbance peak at ≈278 nm for NGQDs and a shoulder around the same region for RGQDs arise from n‐π* transitions of the C = O groups. The characteristic peak ≈300 nm in the absorption spectra of NGQDs can be attributed to the π‐π* transition of C = N bonds.^[^ ^25^ ^]^ The abundance of oxygen functional groups, evident from the FTIR and UV‐Vis spectra, is responsible for high GQD water solubility (Figures 1g,h and 2a). Both GQD types exhibit broad intrinsic fluorescence in the visible with the peaks at ≈500 nm for NGQDs and at ≈530 nm for RGQDs with 400 nm excitation (Figure 2b). This visible emission is attributed to quantum confinement within a plethora of GQD sizes and structures.^[^ ^25^ ^]^

**Figure 3:**
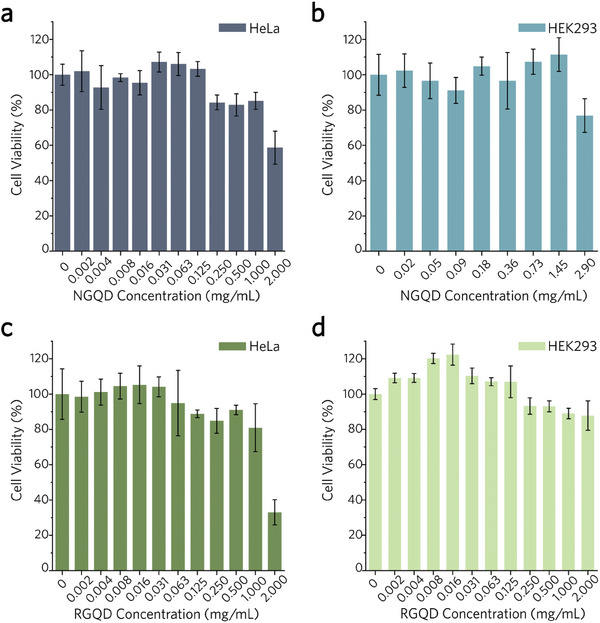
a) Cell viability NGQDs (blue) in HeLa and b) HEK‐293 cells. c) Cell viability of RGQDs (green) in HeLa and d) HEK‐293 cells as evaluated via MTT assay.

MTT assays are further used for the assessment of maximum non‐toxic concentration for cell uptake studies in both HeLa and HEK‐293 cell lines (**Figure** **3**). Despite the high concentrations used GQDs display remarkable cell viability (>80%), at 0.5 – 1.0 mg mL^−1^ in both cell lines. The increase in cell viability above 100% can be explained by the partial degradation of the GQDs in cell environments.^[^ ^24^ ^]^ Consequently, internalization studies employ biocompatible concentrations of 1.0 mg mL^−1^ for NGQDs and 0.5 mg mL^−1^ for RGQDs yielding intracellular fluorescence substantial for quantitative analysis of GQD internalization.

### 2.2 General Understanding of Endocytosis Paths and Inhibitor Functions

Herein, we aim to provide a holistic approach to understanding the potential GQD entry pathways utilizing the six most common chemical endocytosis inhibitors at non‐toxic concentrations,^[^ ^36^, ^58^, ^59^, ^63^ ^]^ each with different mechanisms of action with a total of ≈100 cells analyzed per inhibitor. Since all inhibitors were dissolved in DMSO for consistency, instead of a blank control group, cells were treated with DMSO of the same volume to account for its possible effects on cell population. Before the introduction of the inhibitors, the internalization pathways of each cell line need to be discussed. As displayed in **Table** **2**, both HeLa and HEK‐293 allow for internalization through CME, FEME, and Macro, whereas only HeLa possesses the Cav pathway.^[^ ^70^ ^]^ Uptake through lectin‐dependent endocytosis, CLIC/GEEC, is not found in the HeLa cell line, while some conflicting results are reported about its presence within HEK‐293.^[^ ^71^, ^72^, ^73^ ^]^ The fluorescence of genistein, filipin, and chlorpromazine inhibitors at the concentrations used in the experiment provides negligible contribution that does not affect fluorescence analysis performed in this work. (Figure S5, Supporting Information).

**Table 2:** Internalization paths present in HeLa and HEK‐293 cell lines. (✓) present, (✗) the lack of key molecular component, (○) conflicting evidence.

Cell line	Internalization paths				
	CME	FEME	Cav	CLIC/GEEC	Macro
HeLa	✓	✓^[^ ^74^ ^]^	✓^[^ ^70^ ^]^	✗^[^ ^72^, ^73^ ^]^	✓
HEK‐293	✓	✓^[^ ^75^ ^]^	✗^[^ ^76^ ^]^	○^[^ ^71^ ^]^	✓

**Figure 4:**
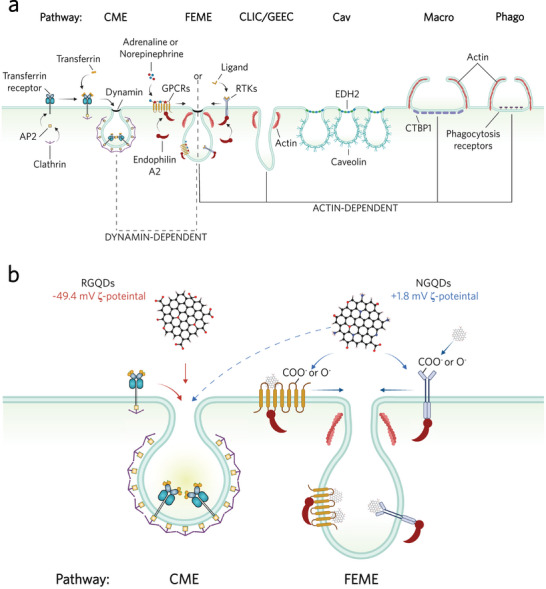
Endocytosis pathways and charge‐dependent uptake of GQDs. a) Schematic representations of endocytosis paths present in both HeLa and HEK‐293 cell lines. These illustrations detail the mechanism, receptors, and their ligands, utilized for each endocytosis pathway. b) Depiction of potential mechanism of action of receptor‐mediated endocytosis pathways (CME and FEME) displaying the effects of GQD charge. Negatively‐charged RGQDs exhibit affinity toward CME, while amine groups of NGQDs are attracted to negatively‐charged functional groups of FEME receptors. Dashed blue lines indicate the secondary preference of NGQDs for CME.

The first inhibitor studied in the present work, cytochalasin D, is commercially deemed a Macro and Phago target inhibitor (Table 1). Its mechanism of action, however, involves depolymerization of F‐actins, a process that not only impacts its intended targets but also plays a crucial role in other pathways that utilize actins in general (**Figure** **4**a).^[^ ^77^, ^78^ ^]^ A careful interpretation of inhibition results is necessitated upon this broad influence when considering the internalization of GQDs in the presence of cytochalasin D. Given the size of GQDs is below the typical threshold necessary to trigger Macro (Figure 1c,f), attributing inhibition observed by the introduction of cytochalasin D solely to this pathway could be inaccurate. It is more plausible that GQDs utilize a combination of internalization pathways such as FEME and CLIC/GEEC, which are susceptible to actin depolymerization,^[^ ^38^, ^39^ ^]^ on top of CME and Cav, since actin filament plays a role in FEME and CLIC/GEEC vesicle formation.^[^ ^78^ ^]^ Further analysis reveals the complexity of using genistein, a broad‐spectrum tyrosine‐specific protein kinase inhibitor.^[^ ^79^ ^]^ Inhibiting protein kinases is shown to partially suppress Cav. However, it is also known that protein kinases play a role in receptor‐mediated endocytosis ranging from regulation of the vesicle formation to receptor recycling.^[^ ^80^, ^81^ ^]^ For instance, although Macro is not known to be a receptor‐mediated pathway, it also can be inhibited by genistein. Formation of Macro is regulated by growth factor signaling, and growth factors activate their respective receptor tyrosine kinases (RTKs). Therefore, the disruption of tyrosine kinase hampers the process.^[^ ^82^ ^]^ Similarly, filipin interacts with the membrane cholesterol as its action is primarily through the disruption of actin‐binding proteins.^[^ ^83^, ^84^ ^]^ This suggests its potential to inhibit actin‐dependent FEME and CLIC/GEEC pathways (Figure 4a). Filipin is also shown to heavily inhibit the formation of the endophilin assemblies that are crucial to FEME,^[^ ^38^ ^]^ while at high concentrations, filipin can lead to off‐target CME inhibition.^[^ ^83^ ^]^ Amiloride is known to be one of the more specific inhibitors, functioning on the Na ion channels inhibiting Na/H ion exchange. Even so, amiloride is a distinct micropinocytosis inhibitor, and there is evidence that its mechanism of action induces the disassembly of actin stress fibers. Such a process likely leads to the reorganization of the actin cytoskeleton, which, in turn, is shown to influence FEME dynamics.^[^ ^38^, ^85^, ^86^ ^]^ Chlorpromazine is a cationic amphiphilic drug that inhibits receptor recycling. This mechanism of chlorpromazine halts the congregation of adaptor proteins (AP2) and clathrin on endosomal membranes, thereby suppressing the formation of clathrin‐coated pits on the plasma membrane. The important role of AP2 in the construction of the endocytic pit implies that its relocation could perturb a clathrin‐mediated pathway (Figure 4a).^[^ ^87^, ^88^ ^]^ Chlorpromazine also displays non‐target FEME inhibition by endophilin‐positive assembly disruption.^[^ ^38^ ^]^ The sixth inhibitor, sodium azide is renowned for its mechanism of action that interferes with the mitochondrial electron transport chain, causing a rapid intercellular ATP depletion^[^ ^89^ ^]^ and mitochondrial flavoprotein NADH dehydrogenase inhibition.^[^ ^90^ ^]^ Glycolysis inhibition provided by sodium azide allows the study of passive and/or facilitated diffusion of GQDs into the cells. Unlike endocytosis, which is highly energy dependent, both passive and/or facilitated diffusion do not require ATP.^[^ ^33^ ^]^ Therefore, the lack of inhibition upon sodium azide treatment is expected to indicate that GQDs utilize passive and or facilitated diffusion. However, it is important to note that sodium azide broadly inhibits all energy‐dependent pathways, complicating the study of nanomaterial endocytosis due to its lack of selectivity.^[^ ^91^ ^]^ This examination, therefore, highlights the intricate interplay among different endocytosis pathways and emphasizes the importance of multifaceted analysis in inhibitor‐based studies.

**Table 1:** List of chemical endocytosis inhibitors used in the experiment, their mechanisms of action, and their target pathways. (✓) – proposed target pathway, (●) off‐ target pathways of inhibitors.

Inhibitors	Mechanisms of action	Endocytosis Pathways						Evaluations
		CME	FEME	Cav	CLIC/GEEC	Macro	Phago	
Cytochalasin D	F‐ actin depolymerization^[^ ^77^ ^]^	●	●	●	●	✓	✓	F‐Actin depolymerization affects most paths^[^ ^78^ ^]^
Genistein	Tyrosine kinase inhibitor^[^ ^79^ ^]^	●	●	●	●	●	●	Partially inhibits Cav^[^ ^80^ ^]^ Could inhibit other receptor‐mediated endocytosis^[^ ^81^ ^]^ and Macro^[^ ^82^ ^]^
Filipin	Binds to membrane cholesterol and disassociates actin‐binding proteins^[^ ^84^ ^]^	●	✓					Inhibits CME but toxic at high concentrations^[^ ^83^ ^]^
Amiloride	Na ion channel, Na/H ion exchange inhibition and possibly induces the disassembly of actin stress fibers^[^ ^85^, ^86^ ^]^		●			✓		Revealed to inhibit FEME^[^ ^38^, ^86^ ^]^
Chlorpromazine	Inhibits the binding of AP2 and clathrin^[^ ^87^ ^]^	✓	●	●				Inhibits off‐target FEME^[^ ^38^ ^]^
Sodium azide	Inhibits glycolysis and ATP production in cells^[^ ^91^, ^92^ ^]^	✓	✓	✓	✓	✓	✓	Inhibits all energy‐dependent paths

### 2.3 Endocytosis of GQDs and its Possible Dependence on Charge

**Figure 5:**
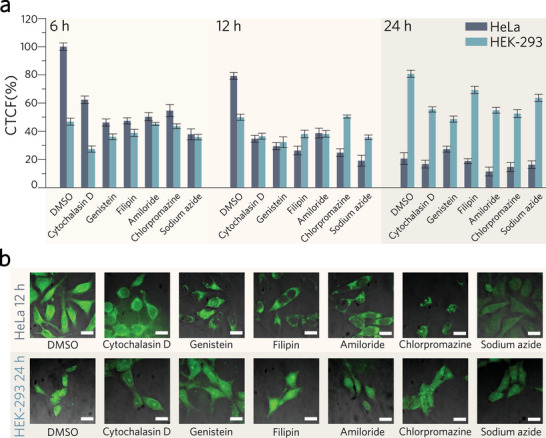
a) Normalized corrected total cell fluorescence (CTCF (%)) of NGQDs in Hela and HEK‐293 cells in the presence of chemical inhibitors at 6, 12, and 24 h. b) Bright‐field/VIS fluorescence confocal overlay images of HeLa cells treated with NGQDs in the presence of chemical inhibitors at 12 h time point. Scale bar = 10 µm.

In order to consider all the specific effects of six major inhibitors, they are applied separately, while GQD internalization into cancer (HeLa) and non‐cancerous (HEK‐293) cells possessing different preferential endocytosis pathways is examined. The primary internalization study is conducted with the NGQDs, while RGQDs are further used to assess the effects of the charge. This strategy permits a comprehensive analysis, where the effect of each inhibitor is elucidated not merely by its target pathway but through its mechanism of action, detailed in Table 1. Non‐internalized GQDs are removed prior to the study by a separate washing step and, thus, do not contribute to observed statistics. Based on TEM size distributions (Figure 1c,f) we expect RGQDs to possess generally greater sizes than NGQDs resulting into somewhat different molarities for different concentrations. However, given that GQDs are a non‐stochiometric material with different sizes and structures and simultaneous internalization of several GQDs could potentially occur we are unable to fully predict the molarity effects on endocytosis while we can evaluate the effects of their charge. Internalization is assessed via intracellular GQD fluorescence calculated in the form of corrected total cell fluorescence (CTCF) (**Figure** **5**a). Highest uninhibited NGQD internalization into HeLa cells takes place within the first 6 h. Due to the relatively low (≈20%) CTCF signal observed, a substantial excretion of NGQDs is expected to take place by the 24th hour, complicating the comparative analysis. Conversely, 24 h corresponds to the maximum internalization of NGQDs in HEK‐293 cells. The introduction of cytochalasin D adds a pronounced inhibitory effect at the 12‐hour mark for NGQDs in both HeLa and HEK‐293 cells. Considering the average 3.65 nm sizes of NGQDs (Figure 1c), Macro is unlikely the inhibited pathway. A more plausible explanation posits the disruption of other receptor‐mediated and actin‐utilizing pathways, affected by cytochalasin D, such as CME, FEME, Cav, and CLIC/GEEC.^[^ ^78^ ^]^ In HEK‐293 cells, with the introduction of cytochalasin D, an analogous drop in fluorescence is observed at all time points as compared to controls, indicating that FEME, CLIC/GEEC, and, possibly, CME pathways still play a role in NGQD endocytosis (Table 1). A similar pattern of inhibition is observed for genistein at 6 h and 12 h: it drastically inhibits internalization into HeLa cells with substantially smaller impact on HEK‐293s. Since genistein inhibits tyrosine kinase, and is shown to inhibit Cav, the lesser inhibition within HEK‐293 compared to HeLa may point out that NGQDs prioritize Cav when it is present (Tables 2 and 1). NGQD endocytosis inhibition caused by genistein at the 24th hour time point in HEK‐293 cells suggests a potential secondary preference toward FEME in that cell line. Filipin causes a significant decrease in NGQD internalization within HeLa, except for 24 h when the excretion occurs, but only negligible inhibition in HEK‐293 cells at all time points. As filipin's mechanism of action, disrupting dynamin by binding to the cholesterol membrane, strongly inhibits FEME and partially – CME, both of these paths are expected to participate in NGQD entry into HeLa cells. At the same time, the absence of such significant inhibition in the HEK‐293 cell line suggests that NGQD internalization could utilize lectins as they proceed through CLIC/GEEC pathways in HEK‐293 cells, especially given that filipin does not inhibit these pathways and HEK‐293 do not possess Cav.

Although not as pronounced as in HeLa, amiloride induces considerable inhibition of NGQD entry into HEK‐293 cells at 12 and 24 h. Since, as previously concluded, NGQDs are not expected to enter through Macro due to their size (Figure 1c), the inhibition of their entry by amiloride suggests their internalization through FEME. Amiloride, which induces actin stress fiber disassembly, is expected to cause off‐target FEME suppression, indicating that both cell lines employ FEME for NGQD internalization (Table 1). Furthermore, the observation of a greater comparative NGQD internalization reduction under amiloride inhibition in HeLa versus HEK‐293 cells strongly suggests FEME‐based NGQD endocytosis in HeLa because amiloride's only off‐target pathway is FEME (Table 1). The second strongest inhibition after sodium azide is generated by chlorpromazine at 12‐hour time point in HeLa cells. This inhibitor halts the binding of AP2 to clathrin, a key component in CME (Figure 4a), which suggests the important role of CME in GQD internalization. However, it is previously observed that chlorpromazine also acts as an off‐target inhibitor for FEME.^[^ ^38^, ^86^ ^]^ Thus, the aforementioned significant inhibition cannot be solely attributed to CME, but, rather, CME, FEME, and Cav combined (Table 1). Finally, the absence of GQD entry inhibition due to chlorpromazine in HEK‐293 at 6 and 12 h can mistakenly lead to the conclusion that CME and FEME do not play a distinct role in the NGQD internalization process for this cell line. However, a substantial inhibition is observed at the 24th hour time point indicating either an uptake due to combination of FEME and CME or just FEME alone. A very general intermediate conclusion can be drawn from the inhibition pattern provided by sodium azide. Given that it inhibits all energy‐dependent pathways, the internalization suppression observed from previous inhibitors and sodium azide conclusively affirms that GQDs internalize through endocytosis. Lesser inhibition caused by sodium azide in HEK‐293 cells compared to HeLa cell line, can indicate the importance of passive and/or facilitated diffusion.

Based on the inhibition effects observed with filipin, amiloride, and chlorpromazine, it is reasonable to infer that NGQDs predominantly enter HeLa cells via FEME and CME. The inhibitory impact of cytochalasin D and genistein further suggests a potential involvement of Cav; however, genistein's lack of specificity precludes a definitive conclusion. For the HEK‐293 cell line, a pronounced preference for lectin‐dependent CLIC/GEEC endocytosis is evident, supporting negligible inhibition under filipin treatment, which typically inhibits FEME and, to a lesser extent, off‐target CME. There is no Cav present in HEK‐293 due to a lack of molecular necessary components, and micropinocytosis can't be considered since its activation requires significantly larger sizes. Chlorpromazine's effect in HeLa cells indicates a marked preference for NGQDs toward CLIC/GEEC endocytosis, as both FEME and CME appear minimally involved. Thus, one can infer that NGQDs prefer FEME in HeLa cells, closely followed by CME with a lesser inclination toward Cav. In HEK‐293 cells, they show a discernible selectivity for CLIC/GEEC endocytosis, with a marginal preference for FEME.

Since GQD internalization into HeLa cells predominantly occurs through receptor‐mediated endocytosis (mainly CME and FEME), this necessitates a deeper exploration of receptor binding mechanisms to elucidate the reasons for such preference. Since both CME and FEME pathways preferred by the GQDs in HeLa cells utilize receptors that interact with charged ligands, the effect of the nanomaterial's charge can play a significant role. During the formation of the FEME vesicles, the SH3 domain of endophilin A2 commonly binds to the compatible Guanine nucleotide‐binding protein‐coupled receptors (GPCRs) such as α_2a_ – and β_1_–adrenergic, dopaminergic D3/D4 receptors, muscarinic acetylcholine receptor 4 or the RTKs, such as EGFR, HGFR, VEGFR, PDGFR, NGFR and IGF1R.^[^ ^38^ ^]^ More than 35% of all FDA‐approved drugs act on GPCRs while 70 other FDA‐approved drugs act on RTKs^[^ ^93^ ^]^ outlining the importance of these pathways. One can speculate that GQDs possessing positively‐charged amine groups can display an affinity toward such endocytic pathways due to the presence of negative hydroxyl groups on ligand‐binding sites of all the GPCRs and RTKs.^[^ ^81^, ^94^ ^]^ Conversely, it can be speculated that the positively charged regions on transferrin encompassing Fe^3+^ions or transferrin receptors utilized in CME can facilitate a simple electrostatic attraction of GQDs that display a substantial negative surface charge. This simplistic description offers a speculatory attempt to explain a part of a more complex mechanism (Figure 4b). For instance, a similar charge‐dependent uptake for RGQDs can also be facilitated through other pathways such as Ca^2+^‐dependent endocytosis.^[^ ^95^ ^]^

**Figure 6:**
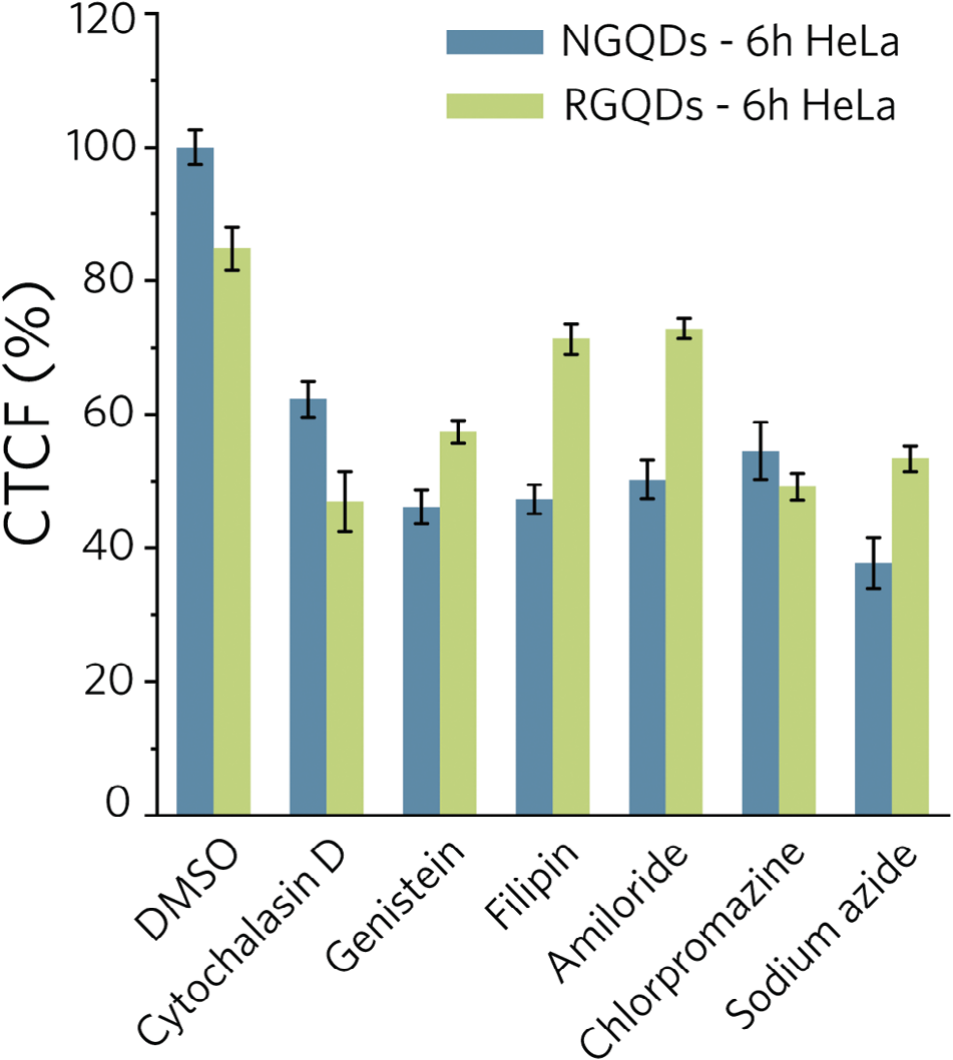
Normalized corrected total cell fluorescence (CTCF (%)) of NGQDs (blue) compared to RGQDs (green) in Hela cells at 6 h.

To experimentally explore charge dependence of GQD endocytosis in HeLa cells, we compare the internalization of negatively charged reduced graphene quantum dots (RGQDs) with a ζ‐potential of ‐49.4 ± 0.4 mV to positively charged NGQDs with a ζ‐potential of +1.8 ± 0.8 mV (Figure S4, Supporting Information) at 6 h (**Figure** **6**). This time point is chosen as it yields maximum NGQD internalization and substantial intake of RGQDs (Figure S8, Supporting Information). In this study, NGQDs show ≈ 20% more internalization under the control DMSO treatment than RGQDs, while with the introduction of the majority of inhibitors, this ratio varies. It is noteworthy that both GQDs exhibit similar internalization inhibition patterns compared to their respective control only under cytochalasin D treatment suggesting that actin plays a significant role in the endocytosis of both nanomaterials with no apparent preference to charge. Genistein's more pronounced inhibitory effect on NGQD internalization, compared to RGQDs, diminishes the likelihood of Cav endocytosis for negatively charged GQDs. The reduced inhibition by filipin and amiloride strongly supports CME as the primary endocytic route for RGQDs, a notion further reinforced by the significant inhibition caused by chlorpromazine. Similarly, to that of NGQDs, RGQD internalization is inhibited when cells are treated with the glycolysis inhibitor sodium azide, a general energy‐dependent path inhibitor, attesting that RGQD internalization is indeed carried out by endocytosis.

## 3 Conclusion

Endocytosis of graphene quantum dots has been previously studied, yet the specific pathways are still debated, particularly due to insufficient consideration of the biological mechanisms of action and potential off‐target effects of inhibitors, resulting in a fragmented understanding and conflicting results in the literature. Our study elucidates the preferential endocytic pathways employed by GQDs in HeLa and HEK‐293 cells, achieved through a comprehensive analysis involving six distinct inhibitors, each characterized by unique mechanistic actions. Fluorescence microscopy‐derived GQD internalization studies indicate that there is not a single path that GQDs prefer, but rather a combination of certain pathways with an apparent hierarchy in their preferences. An intriguing observation is the reduced internalization of NGQDs in HeLa cells treated with genistein, compared to HEK‐293 cells, and suggests a possibility for NGQD internalization via caveolae‐mediated pathways in HeLa cells on top of the more significant FEME and CME preference. However, the non‐specificity of genistein necessitates a cautious interpretation of these findings. In the HEK‐293 cell line, NGQDs primarily utilize the CLIC/GEEC pathways as their initial choice for endocytosis, suggesting the potential importance of lectin dependence. FEME pathway may carry a secondary preference for NGQDs because of a slight amiloride and significant genistein inhibition observed for NGQD internalization in the HEK‐293 cells. Previous studies that are done on GQD derivatives fell short in consideration of the possible non‐target inhibition. Therefore, some works resulted in a single conclusion that endocytosis was either carried through Cav and/or CME. Moreover, most of the previous cell uptake pathway studies did not consider the CLIC/GEEC and FEME as separate pathways while putting them under the clathrin‐independent endocytosis umbrella. However, due to the lack of inhibition observed upon the introduction of sodium azide, one cannot rule out the contribution of passive and/or facilitated diffusion of GQDs into the cells when endocytosis pathways are blocked. Finally, considering a variety of pathways in two cell lines allows this study to predict nanomaterial internalization in different cells in which some pathways are more prevalent than others.

Our findings reveal a charge‐dependent preference in GQD endocytosis through receptor‐mediated pathways. GQD surface chemistry can influence the time spent bound to the cell membrane before internalization. Therefore, successful targeting of receptors that trigger endocytosis can significantly reduce the possibility of provoking cell defense mechanisms such as MPS clearing. Since GQDs in HeLa cells mainly experience receptor‐mediated endocytosis (CME, FEME), as opposed to non‐receptor‐mediated entry (CLIC/GEEC) in HEK‐293, it is safe to assume that there is a higher chance of avoiding MPS clearing while using GQDs for cancer treatment applications. NGQDs are inferred to utilize both FEME and CME in HeLa cells, with a predilection for FEME. We propose that NGQD preference to FEME could arise from the attraction of NGQDs' amine groups to the negatively charged functional groups of FEME receptors formed with GPCRs and RTKs (Figure 4b). This preference is further supported by an earlier (6 h) peak internalization observed in HeLa cells that can be explained by FEME's rapid intake mechanism. Supporting FEME internalization is the observation that in the presence of genistein, a tyrosine kinase inhibitor, NGQDs display less internalization compared to RGQDs (Figure 6). Since RTKs are one of the most common receptors that FEME utilizes (Figure 4a), the drop in internalization upon tyrosine kinase inhibition indicates that NGQDs incline toward FEME as their primary choice of cellular uptake in HeLa. Our results indicate that negatively charged RGQDs predominantly favor CME. Additionally, since HeLa cells used here for RGQD studies do not possess CLIC/GEEC endocytosis vesicles,^[^ ^72^ ^]^ we are not able to conclude on or rule out the possibility of the lectin‐dependent endocytosis for RGQD uptake.^[^ ^96^ ^]^ CME emerges as the secondary internalization pathway for NGQDs possibly due to the presence of a few negatively charged functional groups on their surface. It is important to point out that the difference in the sizes of NGQDs and RGQDs is not expected to affect any internalization parameters as they both reside within the same endocytosis‐relevant size group.

This study challenges the notion of a singular mechanism governing GQD endocytosis, instead proposing a model of preferential pathway usage that varies between cell types and is influenced by the nanomaterial's charge. We previously discussed that both clathrin‐independent paths have their specific mechanisms and targeting FEME necessarily can pave the way for nanomaterials’ usage in clinical studies since it utilizes GPCRs and RTKs, which are present in many of the FDA‐approved drugs. This nuanced understanding of GQD internalization mechanisms not only deepens our comprehension of GQD‐cell interactions but also offers valuable insights for the design of nanomaterials aimed at optimized drug delivery and enhanced efficacy in both *in vivo* and *in vitro* applications.

## 4 Experimental Section

### Synthesis of Top‐Down and Bottom‐Up Graphene Quantum Dots

High porosity RGO (HP‐RGO‐025G, Graphene Supermarket, Ronkonkoma, NY, USA) was oxidized to synthesize RGQDs. The reaction was carried out as follows: RGO was dispersed in deionized (DI) water at 0.25 mg mL^−1^ concentration and 1 mL of sodium hypochlorite (LC246364, LabChem, Zelienople, PA, USA) was added to the suspension. Upon the introduction of NaOCl into aqueous RGO, its decomposition led to the formation of highly reactive oxygen free radicals reacting with RGO graphitic structure, yielding nano‐sized RGQDs. The vials were gently shaken, capped, and left to react for two days in a dark environment under ambient conditions. Products were further purified from unreacted NaOCl and small organic fragments via dialysis in a 1 kDa molecular cutoff dialysis bag (132104, Repligen, Waltham, MA, USA) against DI water for 24 h. For the first 3 h, water in the dialysis chamber was changed every 30 min, after that – every 7 h. After dialysis, the purified suspension was processed through a 0.22 µm syringe filter to remove the unreacted RGO and sterilize the product.

To synthesize NGQDs with a bottom‐up approach, 4 g glucosamine hydrochloride (Sigma‐Aldrich batch #104K0082) was dispersed in 250 mL of DI water and the mixture was microwave‐treated in an HB‐P90D23AP‐ST Hamilton Beach microwave oven for 60 min at 1350 W power. During the microwave treatment, glucosamine underwent a dehydration reaction, leading to its polymerization and formation of NGQDs. After the synthesis, the product was purified from molecular precursors using a 1kDa dialysis bag (132104, Repligen, Waltham, MA, USA) in DI water for 24 h. For the first 3 h, water in the dialysis chamber was changed every 30 min, after that – every 7 h. Both products were further air‐dried to adjust the concentration to 10.5 mg mL^−1^ for RGQDs and to 21.0 mg mL^−1^ for NGQDs.

### Structural and Optical Characterization

The assessment of the nanoscale graphitic structure of the GQDs was performed via HRTEM (high‐resolution transmission electron microscopy, JEOL JEM‐2100). For TEM measurements, the samples were prepared on a carbon‐coated 200 mesh copper grid under ambient conditions. The average size of the GQD structures was derived from TEM analysis, while HRTEM was utilized to ensure their graphitic structure. Infrared absorption was evaluated via the attenuated total reflection (ATR) mode of a Thermo Nicolet Nexus 670 FTIR to identify functional groups present on the GQD surface. Samples for FTIR measurements were freeze‐dried using a Labconco FreeZone 4.5 freeze dryer. In addition to FTIR analysis, surface functional groups of both GQDs are characterized with Kratos Analytical Axis Supra^+^ X‐ray photoelectron spectrometer. Samples were pelletized at 10 000 psi for 30s to form 3mm x 3mm x 1mm pellets for mounting in the XPS instrument and spectra were taken using the Al Kα X‐ray source operating at 220.00 W with an emission current of 15.00 mA. Bruker MultiMode 8 Atomic Force Microscope in tapping mode is used to obtain the height profiles of both GQDs. The UV‐Vis‐NIR absorbance of GQD samples was acquired within the 200 nm range using an Agilent Technologies (Cary 60) absorption spectrometer while their photoluminescence spectra were recorded with Horiba SPEX NanoLog fluorescence spectrofluorometer. ζ Potentials of different GQD suspensions at 1 mg mL^−1^ were measured using a NanoBrook ZetaPALS instrument to reflect their surface charge.

### Cell Culture

In vitro studies were performed using human embryonic kidney (HEK‐293) cells as the model non‐cancer cell line and HeLa cells as the model cancer cell line. The cells were cultured in Dulbecco modified Eagle's medium (D6046, Sigma‐Aldrich) and 10% fetal bovine serum (16140‐063, Gibco) with the addition of 1% L‐glutamine (G7513, Sigma‐Aldrich), the minimum essential medium (MEM), nonessential amino acid solution (M7145, Sigma‐Aldrich) and penicillin/streptomycin (P4333, Sigma‐Aldrich). Cell cultures were kept in an incubator with 5% CO_2_ at 37 ^°^C and used for cell viability assays, cell uptake experiments, and fluorescence microscopy experiments.

### Cell Viability Assay

Standard MTT cell viability assays were employed to assess the biocompatibility of the GQDs. Both HeLa and HEK‐293 cells were plated in a 96‐well plate at a density of 5000 cells per well (100 µL per well) and kept in an incubator overnight at 37.1 ^°^C with 5% CO_2_. After 24 h incubation, the medium was replaced by 100 µL of 1 mg mL^−1^ of thiazolyl blue tetrazolium bromide. After 4 h of further incubation, 3‐(4‐dimethylthiazol‐2‐yl)‐2,5‐diphenyltetrazolium bromide (MTT) was replaced with 100 µL of DMSO (dimethyl sulfoxide) to solubilize the formazan, a highly absorbing, blue‐colored byproduct formed in metabolically active cells. The absorbance of formazan, proportional to cell viability, was measured in each well at 580 nm using a FLUOstar Omega microplate reader.

### Cell Uptake Experiments and Chemical Inhibitors

Both HeLa and HEK‐293 cells were seeded at 1 × 10^4^ cells onto glass coverslips placed in a six‐well plate with 2 mL media in each well and incubated for 24 h at 37.1 ^°^C with 5% CO_2_. Cells were treated with 100 µL each: DMSO for control samples, or 532 µg mL^−1^ Amiloride hydrochloride hydrate, or 142 µg mL^−1^ Chlorpromazine hydrochloride, or 40 µg mL^−1^ filipin complex from *Streptomyces filipinensis*, or 100 µg mL^−1^ cytochalasin D, or 1.081 mg mL^−1^ genistein, or 3.901 mg mL^−1^ sodium azide (NaN_3_) for samples subjected to different endocytosis inhibitors. All inhibitors were bought from Sigma‐Aldrich (USA) in powder form and dissolved in DMSO. Treated cells were left in an incubator for 30 min, sufficient for endocytosis pathway inhibition to take place. Afterward, 100 µL of 21 mg mL^−1^ NGQDs or 10.5 mg mL^−1^ of RGQDs were added to the cells and left to internalize for 6 h, 12 h, and 24 h. Coverslips with cells were further washed with 1× phosphate‐buffered saline (PBS) to remove GQDs that did not internalize. Following the washing step, the cells were fixed with 4% formaldehyde solution (28908, Thermo Scientific) and 1× Fluoromount‐GTM mounting medium (00‐4958‐02, Invitrogen) and sealed onto microscope slides for imaging.

### Confocal Fluorescence Microscopy Imaging

Fluorescence microscopy imaging in the visible was done with a semi‐motorized inverted Olympus IX73 fluorescence microscope with a 60× (IR‐corrected Olympus Plan Apo) water immersion objective coupled to a Photometrics Prime 95B CMOS camera through an Olympus DSU (disk spinning unit) confocal system. Images were taken with 480 ± 20 nm filtered lamp excitation and 535 ± 20 nm emission filter for both GQD types.

### Image Processing

Image J software (1.53a, National Institutes of Health, Bethesda, MD, USA) was utilized for the assessment of the intercellular fluorescence intensity. Corrected total cell fluorescence (CTCF) was calculated to quantify cellular internalization. CTCF is calculated by subtracting the product of the area of the selected cell and the mean fluorescence of background readings from the integrated density of the cell:

(1) $$\begin{array}{ccc} & & \mathrm{CTCF}=\mathrm{Normalized}\;\mathrm{Integrated}\;\mathrm{Density}\\ & & -\left(\mathrm{Area}\;\mathrm{of}\;\mathrm{selected}\;\mathrm{cell}\times \mathrm{The}\;\mathrm{mean}\;\mathrm{fluorescence}\;\mathrm{of}\;\mathrm{background}\;\mathrm{readings}\right) \end{array}$$

Normalized Integrated Density is defined as the mean value of the pixel brightness in the selected cell multiplied by the total area of the cell. The mean fluorescence of the background reading is represented as the normalized integrated density of the background per unit area.

Initially regions of interest (ROI) were obtained by outlining each individual cell (≈ 100 cells per treatment at one time point for each cell line and nanomaterial. In total >4000 cells for NGQDs and >2000 cells for RGQDs are outlined and analyzed). Intracellular fluorescence is reflected in the Integrated Density, the mean gray value for each pixel within the selected ROI. Later, to account for background fluorescence signal, Integrated Density of an empty area the size of a cell is subtracted from the Integrated Density of each cell. In order to inter‐compare the results, all fluorescence data within Figure 5 and Figure S7 (Supporting Information) is normalized to the corresponding highest internalization time point, 6 h for NGQDs and 12 h for RGQDs respectively.

Confocal overlay images (Figures 5b and S6 and S7b, Supporting Information) were obtained by overlaying fluorescence images with bright field images in Image J software.

## Conflict of Interest

The authors declare no conflict of interest.

## Supporting information



Supporting Information

## Data Availability

The data that support the findings of this study are available from the corresponding author upon reasonable request.
