# Supplementary material for: Holistic Investigation of Graphene Quantum Dot Endocytosis
Source: Small. 2025 Feb 2;21(9):2406095. doi: 10.1002/smll.202406095 (PMC11878264; doi:10.1002/smll.202406095)
Supplement: Supplementary file 1 — Supporting Information [file SMLL-21-2406095-s001.docx]

Supporting Information


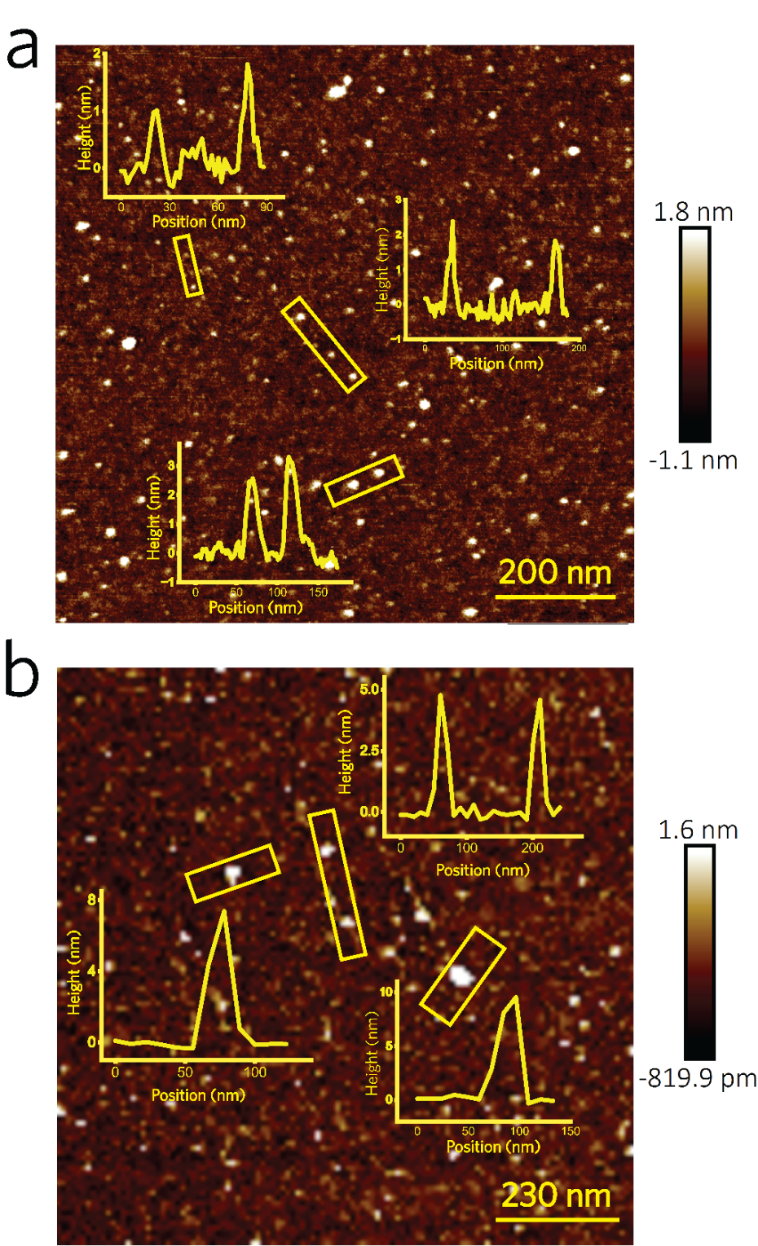


**Figure S1.** AFM height profiles for a) NGQDs and b) RGQDs.


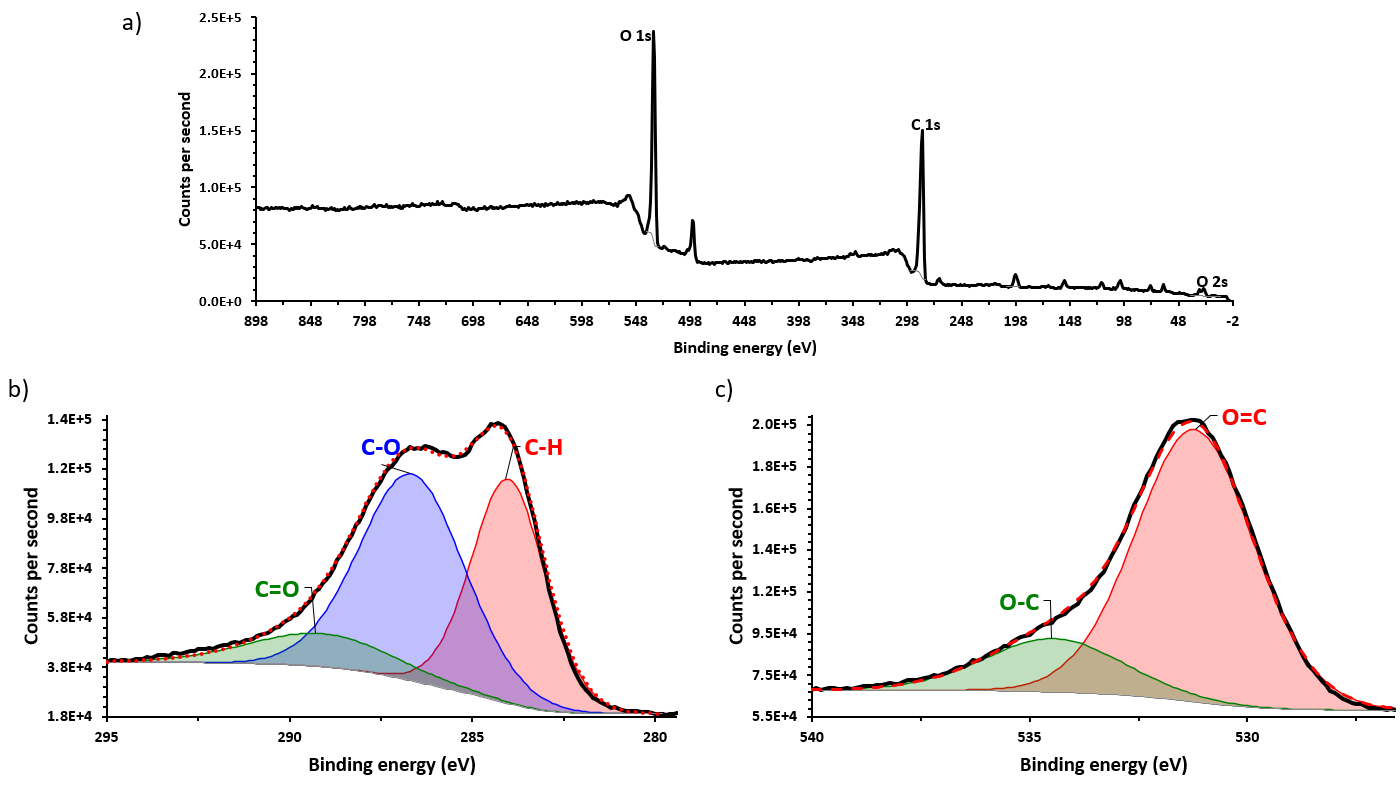


**Figure S2.** (a) XPS survey spectrum of RGQDs (b) Deconvoluted C1s XPS spectra of RGQDs from Voigt fit with Shirley background correction (c) Deconvoluted O1s XPS spectra of RGQDs from Voigt fit with Shirley background correction


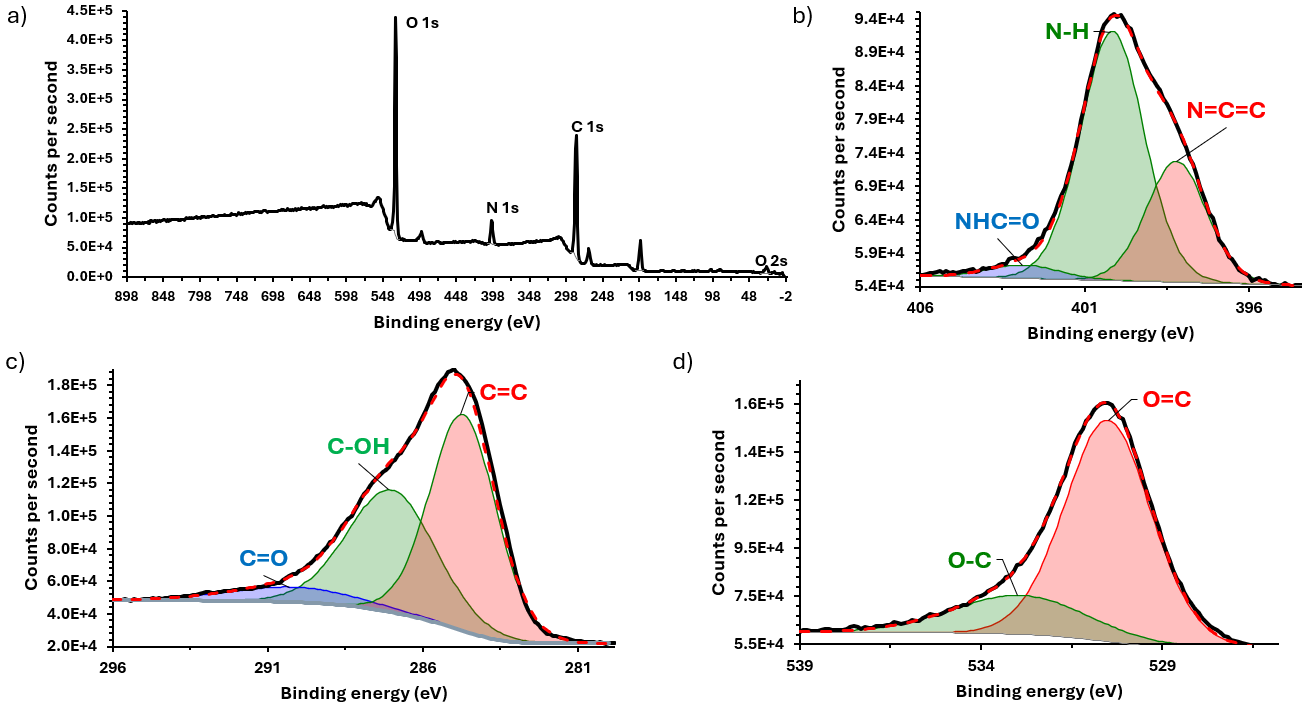


**Figure S3.** (a) XPS survey spectrum of NGQDs (b) Deconvoluted N1s XPS spectra of RGQDs from Voigt fit with Shirley background correction (c) Deconvoluted C1s XPS spectra of RGQDs from Voigt fit with Shirley background correction (d) Deconvoluted O1s XPS spectra of RGQDs from Voigt fit with Shirley background correction


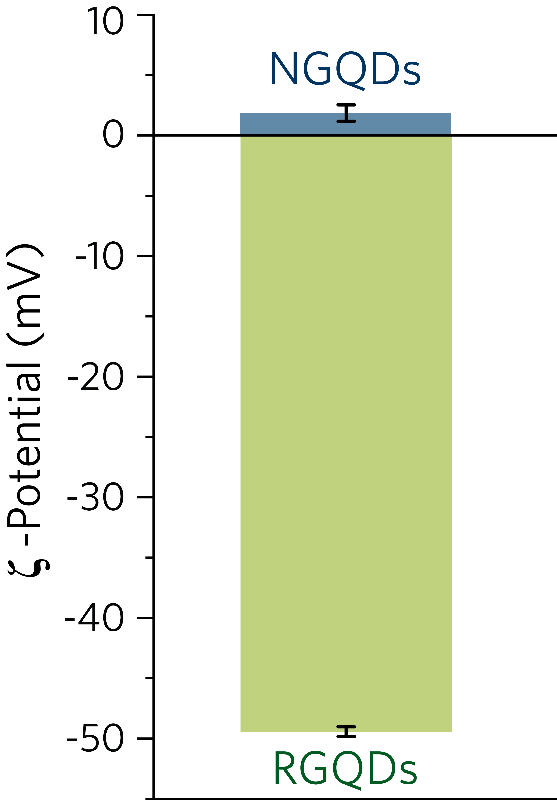


**Figure S4.** ζ-potentials of NGQDs (blue) and RGQDs (green) at pH 7.


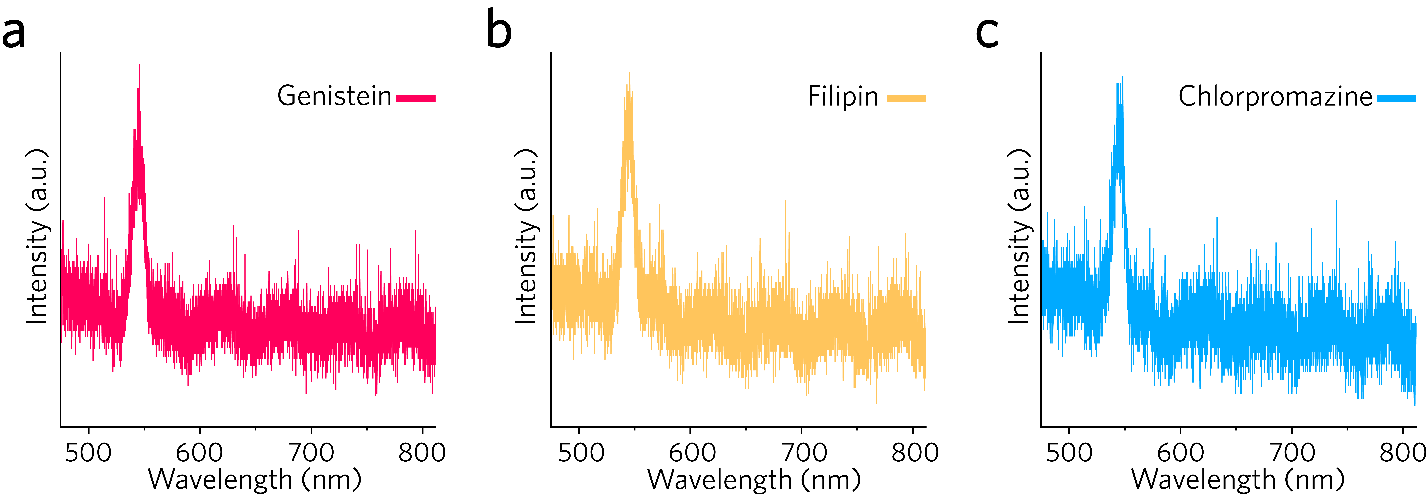


**Figure S5.** Emission spectra of a) genistein (red), b) filipin (yellow) and c) chlorpromazine (blue) measured at 460 nm excitation at the concentrations used for the experiment. Spectra displays that these three inhibitors do not contribute to the fluorescence under 460 nm excitation.


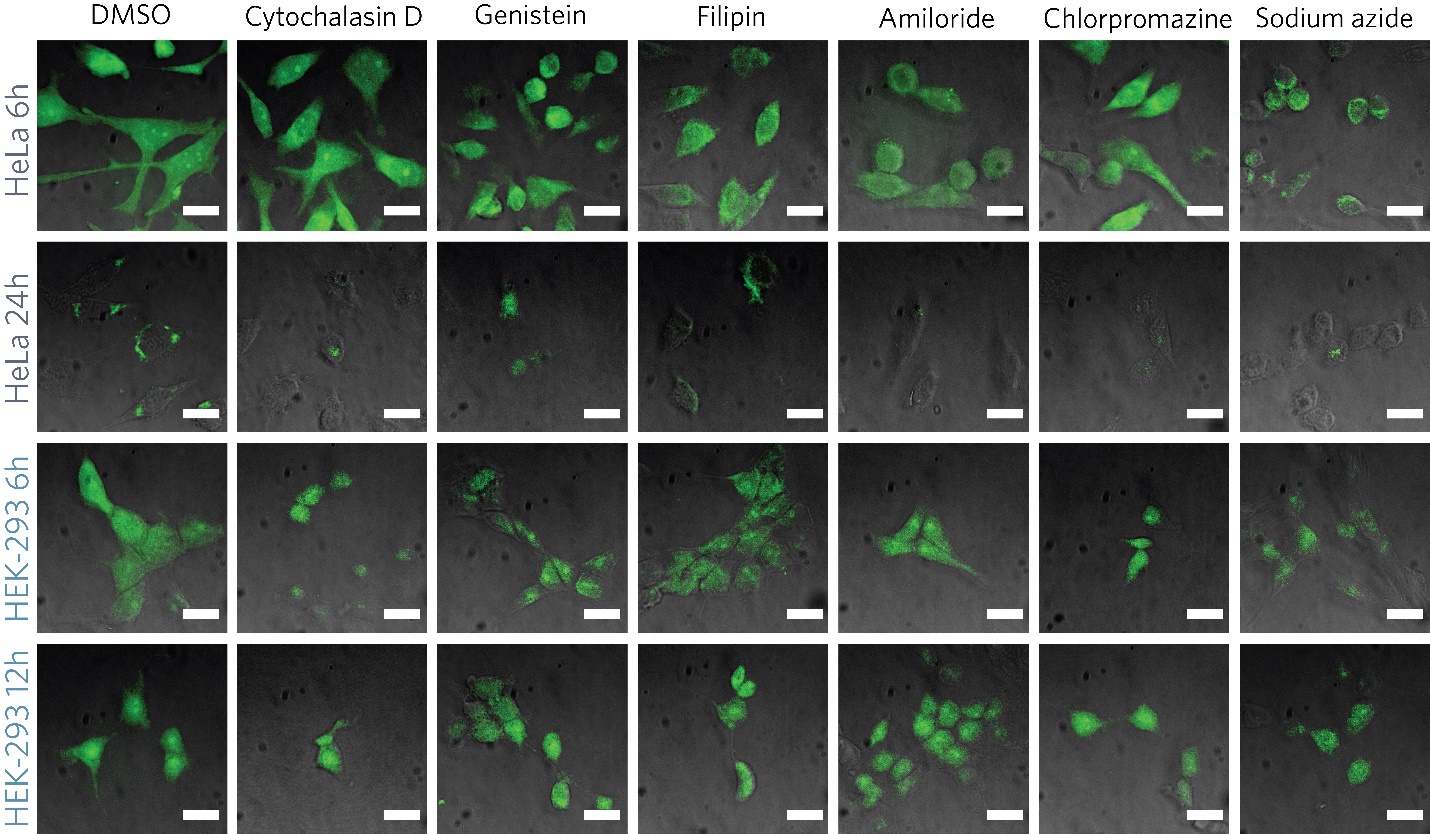


**Figure S6.** Bright-field/VIS fluorescence confocal overlay images of HeLa cells at 6 and 24h, HEK293 cells at 6 and 12h treated with NGQDs in the presence of chemical inhibitors. Scale bar = 10 μm.


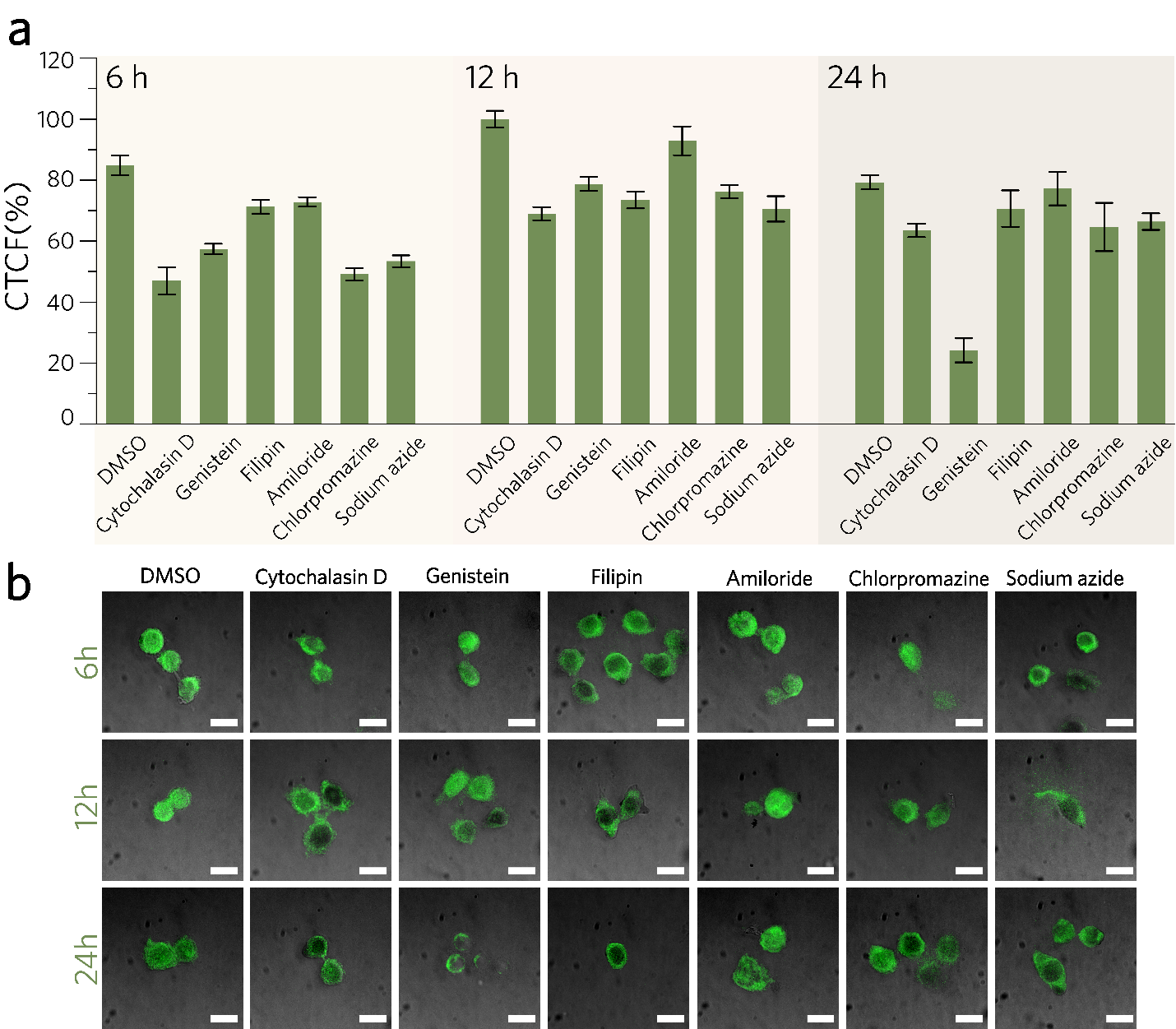


**Figure S7.** a) Normalized corrected total cell fluorescence (CTCF (%)) of RGQDs in Hela in the presence of chemical inhibitors at 6, 12 and 24 h. b) Bright-field/VIS fluorescence confocal overlay images of HeLa cells treated with RGQDs in the presence of chemical inhibitors at 6, 12 and 24 h. Scale bar = 10 μm.


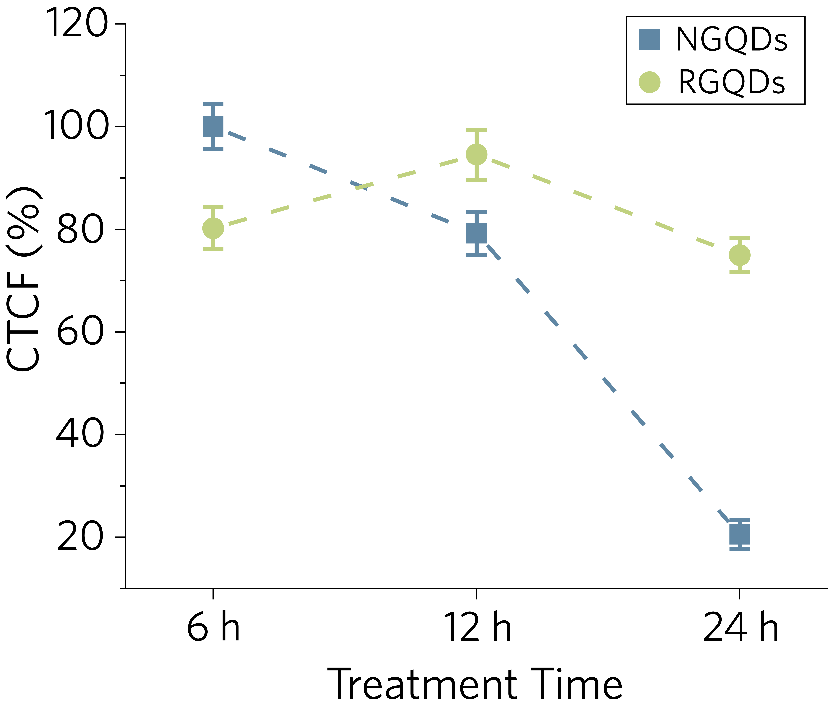


**Figure S8.** Comparative internalizations of NGQDs and RGQDs in HeLa cells with DMSO treatment (control group) at 6, 12 and 24 h. Corrected total cell fluorescence (CTCF (%)) was normalized to the highest fluorescence value observed (NGQD at 6h) to assess the comparative internalizations of NGQDs and RGQDs when no inhibitors are present.
